# Interoception in Female Adolescents with Inflammatory Bowel Diseases *Versus* Restrictive Eating Disorders

**DOI:** 10.3390/nu18020251

**Published:** 2026-01-13

**Authors:** Anna Riva, Gabriele Arienti, Carlo Panarella, Eleonora Brasola, Simona Di Guardo, Giovanna Zuin, Laura Spini, Naire Sansotta, Andrea Eugenio Cavanna, Renata Nacinovich

**Affiliations:** 1 Department of Child Neuropsychiatry, Fondazione IRCCS San Gerardo dei Tintori, 20900 Monza, Italy; anna.riva@irccs-sangerardo.it (A.R.); gabriele.arienti@irccs-sangerardo.it (G.A.); carlo.panarella@irccs-sangerardo.it (C.P.); eleonora.brasola@irccs-sangerardo.it (E.B.); simona.diguardo@irccs-sangerardo.it (S.D.G.); l.spini4@campus.unimib.it (L.S.); 2 Department of Pediatrics, Fondazione IRCCS San Gerardo dei Tintori, 20900 Monza, Italy; giovanna.zuin@irccs-sangerardo.it; 3 School of Medicine and Surgery, University of Milano-Bicocca, 20900 Monza, Italy; a.e.cavanna@bham.ac.uk; 4 Department of Paediatric Hepatology Gastroenterology and Transplantation, Papa Giovanni XXIII Hospital, 24127 Bergamo, Italy; nsansotta@asst-pg23.it; 5 Department of Neuropsychiatry, National Centre for Mental Health, Birmingham and Solihull Mental Health NHS Foundation Trust, Birmingham B15 2FG, UK; 6 School of Medical Sciences, College of Medicine and Health, University of Birmingham, Birmingham B15 2TT, UK; 7 School of Life and Health Sciences, Aston Brain Centre, Aston University, Birmingham B4 7ET, UK; 8 Sobell Department of Motor Neuroscience and Movement Disorders, Institute of Neurology and University College London, London WC1N 3BG, UK

**Keywords:** IBDs, restrictive eating disorders, GBA, female, adolescents, interoception

## Abstract

**Background:** Female individuals with inflammatory bowel diseases (IBDs) are more likely to develop restrictive eating disorders (REDs), with both conditions appearing to share common pathophysiological pathways. We conducted a case–control study exploring eating symptomatology and interoceptive profiles in female adolescents with IBDs compared with adolescents diagnosed with REDs, in order to test the hypothesis that the two clinical populations exhibit similar interoceptive characteristics. **Methods:** We recruited 33 female adolescents with IBDs and 54 controls with REDs matched for age and gender. All participants completed a validated psychometric battery assessing eating disorder features (EDI-3) and interoceptive awareness (MAIA-2). **Results:** Twenty-seven percent of patients with IBD scored above the cut-off (>70th percentile) on the EDI-3 Eating Disorder Risk Composite (EDRC), showing an eating and interoceptive profile comparable to that of patients with REDs. The two sub-cohorts within the IBD sample differed in the ‘Not-Worrying’ and ‘Trusting’ MAIA-2 subscales, with the IBD cohort at risk of developing an ED reporting lower scores. **Conclusions:** Our findings indicate comparable interoceptive profiles between adolescents with IBDs who are at risk of developing EDs and patients with a confirmed diagnosis of REDs. This similarity underscores the need to further investigate the shared pathogenic mechanisms underlying these conditions, particularly the role of the gut–brain axis (GBA).

## 1. Introduction

Inflammatory bowel diseases (IBDs) are severe and debilitating chronic gastrointestinal disorders that may affect children and adolescents and include Crohn’s disease (CD), ulcerative colitis (UC), and IBD unclassified (IBD-U) [1]. Onset during childhood typically occurs in approximately 25% of cases [2]. Recent studies [3,4,5,6] suggest a link between gastrointestinal conditions, such as IBDs, and a range of psychiatric disorders [7], with evidence indicating that disturbances in the gut–brain axis (GBA) may contribute to IBD pathophysiology [8].

Psychological and psychiatric problems in both children and adults affected by IBDs are very common [9], but often remain undiagnosed [10,11]. In a recent case–control study, adolescents with IBDs reported more severe symptoms across all areas of psychopathology [12]. Further, results of international paediatric cohort studies [5,6,13] confirmed that receiving a diagnosis of IBD increase the risk of several psychiatric disorders as affective and anxiety disorders, post-traumatic stress disorders (PTSDs) and eating disorders (EDs).

Restrictive eating disorders (REDs) [14] are severe conditions associated with both medical [15,16] and psychiatric co-morbidities [17]. It has been suggested that IBDs and EDs could have overlapping pathogenic pathways, as well as shared genetic, environmental, and psychological factors. Specific clinical features (i.e., disease relapses) increase the risk of developing both EDs and alexithymia in adolescents with IBDs [18]. Recent research conducted by our group outlined common psychological traits between adolescents with REDs and those with IBDs at risk of developing EDs [19].

Interoception has been defined as “the process by which the nervous system senses, interprets, and integrates signals originating from within the body, providing a moment-to-moment mapping of the body’s internal landscape at both conscious and unconscious levels” [20]. Recent conceptualizations of interoception include internally originating signals—e.g., visceral signals, heartbeat, respiratory rate, as well as representations of skin and body temperature, pain, itch, and sensory touch [21,22].

Interoceptive deficits have long been recognized as a key feature of EDs [23], as they have been observed across different phenotypes and are currently considered a transdiagnostic feature [24,25]. Furthermore, altered interoception has been described in subclinical populations at risk of developing EDs and appears to persist after recovery from the illness [26,27]. Albeit scarce and limited to adult populations, studies investigating interoceptive functions and their role in emotional experience in individuals with somatic disorders such as IBDs suggest that specific interoceptive alterations may be detected [28].

Based on previous research, we hypothesized that female adolescents with REDs and female adolescents with IBDs at risk of developing EDs share common interoceptive characteristics. The main aim of this study was to examine the interoceptive profiles of female adolescents with IBDs in comparison to age- and gender-matched peers with REDs, with the hypothesis that adolescents with REDs and those with IBDs at risk of developing eating disorders exhibit similar interoceptive features. Our secondary objective was to explore potential associations between the socio-demographic and clinical characteristics (including longer disease duration and exposure to steroid cycles) and interoceptive profiles in patients with IBDs at risk of developing EDs, in order to evaluate the potential influence of disease-related features on interoception.

Gender-specific differences in IBDs have been reported across multiple domains, including epidemiology, clinical presentation, disease course, treatment response, and psychosocial functioning. Although these differences are generally less pronounced than in other autoimmune conditions, they underscore the importance of considering sex when investigating disease mechanisms and patient outcomes [29]. Similarly, REDs are considerably more prevalent in females during adolescence, while emerging evidence indicates that male adolescents may exhibit distinct psychological and behavioural profiles, including higher rates of purging, excessive exercise, and elevated anxiety and obsessive–compulsive traits [30]. Collectively, these observations informed our choice to focus the present study on female adolescents, thereby enhancing sample homogeneity, reducing gender-related variability, and limiting potential confounding factors.

## 2. Materials and Methods

### 2.1. Study Participants and Ethics Statement

Between October 2022 and April 2024, 83 female adolescents aged 13–18 years were enrolled from two distinct clinical cohorts: a sample of 33 patients with a confirmed diagnosis of IBDs for at least six months, established through a combination of endoscopic, radiological, biochemical, and histological assessments in line with the ESPGHAN Revised Porto Criteria [1], and a sample of 54 adolescents with a confirmed diagnosis of REDs [14] confirmed using a semi-structured DSM-5 interview [31]. The IBD patients were recruited from the Pediatric Gastroenterology Unit, Department of Pediatrics, Fondazione IRCCS San Gerardo (Monza, Italy), and the Department of Pediatric Hepatology, Gastroenterology, and Transplantation, Papa Giovanni XXIII Hospital (Bergamo, Italy). The REDs sample was recruited from the Eating Disorders Unit, Department of Child Neuropsychiatry, Fondazione IRCCS San Gerardo (Monza, Italy). The RED sample was recruited at the time of the first assessment at the Eating Disorders Unit to minimize the potential influence of therapeutic interventions for EDs on the test results.

All adolescents and their parents received comprehensive information regarding the aims of the study, and written informed consent was obtained from the parents of all participants. Ethical approval was granted by the Brianza Ethics Committee (Protocol code: 311, 29 June 2023), in compliance with the principles of the Declaration of Helsinki (1964) and its subsequent revisions.

### 2.2. Criteria for Exclusion

The exclusion criteria for patient enrolment were as follows: presence of diagnosed psychiatric co-morbidities (including EDs) in patients with IBDs, intellectual disabilities and insufficient comprehension of the Italian language for all subjects. All the recruited subjects completed the full set of self-report questionnaires and were included in the study.

### 2.3. Assessment Tools

Demographic information and disease-related clinical data, including Body Mass Index (BMI, weight (kg)/height (m)^2^) at the time of assessment, as well as the specific types of IBDs and REDs, were collected for all participants. For participants in the IBD cohort, additional data were obtained, including current pharmacological treatments, total number of disease relapses and in the previous six months, steroid treatment cycles (both cumulatively and in the preceding six months), and number of hospitalizations.

Moreover, all participants completed a standardized psychometric battery assessing eating disorder symptoms [32] and interoceptive awareness [33].

The Eating Disorders Inventory-3 (EDI-3) is a self-report questionnaire that evaluates psychological traits and symptomatology relevant to individuals with EDs. It comprises 91 items distributed across 12 primary scales, which are further combined into six composite scores: one specific to eating disorder risk (Eating Disorder Risk) and five assessing broader psychological constructs (Ineffectiveness, Interpersonal Problems, Affective Problems, Overcontrol, and Global Psychological Maladjustment). The Italian adaptation of the EDI-3 has shown strong internal consistency (Cronbach’s α = 0.80–0.90) and excellent test–retest reliability (r = 0.93–0.98). In the present study, analyses focused on the six composite scores [32].

The MAIA-2 (Multidimensional Assessment of Interoceptive Awareness-2) [33] is a self-report questionnaire evaluating multiple dimensions of interoception. It contains 37 items, each rated on a six-point Likert scale ranging from 0 (never) to 5 (always). Scores for each subscale are computed as the mean of the items within that scale, with higher scores reflecting more adaptive self-reported interoceptive abilities. The questionnaire includes the following eight dimensions: (1) Noticing (“awareness of uncomfortable, comfortable, and neutral body sensations”); (2) Not-Distracting (“tendency not to ignore or distract oneself from sensations of pain or discomfort”); (3) Not-Worrying (“tendency not to worry or experience emotional distress with sensations of pain or discomfort”); (4) Attention Regulation (“ability to sustain and control attention to body sensations”); (5) Emotional Awareness (“awareness of the connection between body sensations and emotional states”); (6) Self-Regulation (“ability to regulate distress by attention to body sensations”); (7) Body Listening (“active listening to the body for insight”); (8) Trusting (“experience of one’s body as safe and trustworthy”). Cronbach’s alpha scores for the eight subscales range from 0.64 to 0.83. The present study was conducted using the Italian version of the MAIA-2.

### 2.4. Data Analysis

Means and standard deviations (S.D.) were used to summarize continuous variables, while categorical variables were reported as absolute or relative frequencies. As the data did not meet normality assumptions, non-parametric methods were employed. In the IBD cohort, participants scoring above the 70th percentile on the EDI-3 EDRC subscale, which represents the threshold for pathological values, were compared with those below the cut-off and with adolescents diagnosed with REDs. Comparisons of socio-demographic characteristics, disease-related measures, EDI-3 composite scores, and MAIA-2 subscales across the three cohorts were conducted using the Kruskal–Wallis one-way ANOVA. Spearman’s correlation analyses were performed to explore relationships between disease parameters and MAIA-2 scores. All statistical analyses were conducted using IBM SPSS Statistics (version 29.0.1.0).

## 3. Results

We analysed data from 87 female adolescents aged 13 to 18 years: 33 adolescents with IBDs and 54 adolescents with REDs. Table 1 provides a summary of the socio-demographic and clinical characteristics of the two cohorts. In the cohort of adolescents with IBDs, UC was the most frequent diagnosis (66.7%), whereas in the RED cohort, anorexia nervosa (AN) was the most common diagnosis (62.9%). The two cohorts differed for BMI at evaluation (*p* < 0.001).

The clinical characteristics of the IBD cohort are reported in Table 2.

Within the sample of adolescents affected by IBDs, 9/33 (27%) were found to be at risk of developing EDs based on their scores on the composite EDRC subscale of the EDI-3 (EDI-EDRC>70th percentile). The results of comparisons of age and BMI at evaluation among the three clinical cohorts (IBD-EDRC>70 *versus* IBD-EDRC<70 *versus* REDs) are summarized in Table 3. Patients with REDs differed from the two IBD cohorts in BMI. Patients in the IBD-EDRC>70 cohort were older than patients with IBD-EDRC<70.

Table 4 summarizes the results of the comparison between the three cohorts with regard to the EDI-3 composite scales. The RED cohort and the IBD-EDRC>70 cohort did not differ on any of the EDI-3 composite scales. In contrast, there were significant differences across all scales between the IBD-EDRC<70 cohort and the IBD-EDRC>70 cohort, as well as between the IBD-EDRC<70 cohort and the RED cohort. The only exception was the EDI-IPC scale, for which no difference was observed between the two IBD cohorts

Table 5 summarizes the results of the comparisons for the MAIA-2 scale. The IBD-EDRC>70 cohort showed intermediate scores between the other two cohorts in most MAIA-2 subscales, with the RED cohort reporting the lowest scores. The RED cohort and the IBD-EDRC>70 cohort did not show statistically significant differences on any of the MAIA-2 subscales. In contrast, the IBD-EDRC<70 cohort reported higher scores than the IBD-EDRC>70 cohort on the MAIA-2 ‘Not Worrying’ (*p* < 0.05) and ‘Trusting’ (*p* < 0.004) subscales. Compared with the RED cohort, the IBD-EDRC<70 cohort reported higher scores on the MAIA-2 ‘Not Worrying’ (*p* < 0.002), ‘Attention Regulation’ (*p* < 0.001), ‘Self-Regulation’ (*p* < 0.001), ‘Body Listening’ (*p* < 0.001), and ‘Trusting’ (*p* < 0.001) subscales.

Table 6 summarizes the correlations between the main clinical disease characteristics and the MAIA-2 subscale scores. No statistically significant correlations were observed, except for indirect correlations between MAIA-2 ‘Attention Regulation’ and disease duration (*p* < 0.037), and between MAIA-2 ‘Self-Regulation’ and both the number of steroid cycles in the preceding six months (*p* < 0.046) and disease duration (*p* < 0.018).

## 4. Discussion

Children and adolescents with IBDs may be at an increased risk of developing EDs, potentially due to shared underlying biological mechanisms [18]. Further, alterations in the GBA have been implicated in the pathophysiology of both EDs [34,35,36] and IBDs [4]. This hypothesis is consistent with observations that adolescents with IBDs and adolescents with REDs share common psychological features, including drive for thinness, interpersonal insecurity, anxiety, depression, and alexithymia [19]. The present study focused on the role of interoception as a possible risk factor for the development of EDs and for their persistence after recovery [25,27] in female adolescents with IBDs at risk of developing EDs.

In our sample, more than half of the adolescents with IBDs had UC (61.8%), and more than half of those with REDs had typical AN (62.9%), in line with epidemiological data [26]. Within the IBDs sample, 27% of patients screened positive for the risk of developing EDs (EDI-3 EDRC>70th percentile). These findings are higher than the 2.7% prevalence of eating disorders reported among adolescents in the general population [37] and are higher than the 5–17% risk generally observed in patients with IBDs [19,38,39]. This result could be related to the previous observation that the risk of developing EDs in adolescents with IBD is higher in females than in males [18].

Patients with IBDs at risk of developing EDs (EDI-EDRC>70 cohort) were characterized by EDI-3 profiles concerning eating symptomatology overlapping with those of patients with REDs, and significantly different from those of the IBD cohort without risk of developing disordered (EDI-EDRC<70 cohort). These findings are consistent with previous literature reporting overlapping psychological features between adolescents with IBDs and those with REDs [19].

Similarly, the EDI-EDRC>70 cohort reported MAIA-2 scores characterized by interoceptive profiles overlapping with those of the RED cohort, with mostly lower scores (and higher deficits) than the EDI-EDRC<70 cohort. In particular, patients in the EDI-EDRC>70 cohort reported lower scores (corresponding to higher deficits) than the EDI-EDRC<70 cohort on the ‘Not-Worrying’ and ‘Trusting’ MAIA-2 subscales. These findings are particularly relevant because the ‘Not-Worrying’ subscale assesses the tendency not to worry or experience emotional distress in response to sensations of pain or discomfort. Abdominal pain is a highly debilitating symptom in patients with IBDs, which may persist despite inflammation control and can be partially managed through diet and nutrition [40]. These results suggest that adolescents with IBDs at risk for developing EDs are more concerned about pain sensations than those without such risk: in turn, this may lead to more restrictive dietary habits and further increase the risk of developing EDs. Another notable difference concerns the ‘Trusting’ subscale, which measures the experience of one’s body as safe and trustworthy. A recent network analysis [41] indicated that the sensation of being unsafe in one’s body represents the strongest connection between ED symptoms and the body awareness dimension in female. The authors proposed that reduced body trust constitutes a central aspect of interoceptive awareness in individuals with EDs. In our sample, a statistically significant difference on this subscale was observed between the two IBD cohorts, with lower scores in the EDI-EDRC>70 cohort. This finding supports the hypothesis that the interoceptive profile of adolescents with IBD at risk of developing EDs resembles that of the RED cohort.

A further result of our study suggests the absence of significant correlations between disease features and interoceptive profiles in adolescents with IBDs at risk of developing EDs, indicating that the interoceptive profile may not be dependent on disease course. Although this finding is intriguing and is in line with the hypothesis of a shared psychopathological core between the two disorders, it should be interpreted with caution due to the limited statistical power of our sample. Although this study provides novel insights, it presents some limitations. In particular, the small sample size in particular of the IBD group may have reduced statistical power, potentially limiting the detection of significant associations with specific disease characteristics and constraining the generalizability of the findings. Moreover, further research should be conducted by including a control group in order to validate our findings. Another limitation is the use of sole self-report measures, particularly as both healthy adolescents and clinical populations may show deficits in self-awareness. Patients with IBDs diagnosed with comorbid mental disorders were excluded to focus on a more homogeneous sample: this choice may have led to an underestimation of the actual prevalence of mental health problems in this population and may have limited the clinical representativeness of our sample. Finally, potential referral bias must be acknowledged, as participants with EDs or IBDs were recruited from tertiary care centers, which typically manage more severe or complex cases; therefore, our sample may not fully represent the broader population of adolescents with REDs or IBDs.

## 5. Conclusions

The current study, although exploratory and cross-sectional in nature, revealed significant similarities in interoceptive profiles between female adolescents with IBDs at risk for EDs and those with a confirmed diagnosis of REDs. If replicated in larger studies, these findings would strengthen the evidence for an association between IBDs and REDs, emphasizing the importance of further investigating the shared pathogenic mechanisms underlying both conditions—particularly the role of the GBA. Importantly, these results have practical implications, including the potential for earlier identification of at-risk adolescents, the development of targeted preventive strategies, and the tailoring of interventions to address interoceptive deficits, with the aim of improving both psychological and gastrointestinal outcomes during adolescence. Future research should also aim to develop more effective treatment approaches for individuals with IBDs who are vulnerable to developing EDs, with particular attention to the female adolescent population.

## Figures and Tables

**Table 1:** Socio-demographical and clinical characteristics of female adolescents with IBD and REDs.

	IBDs (N = 33)	REDs (N = 54)	*p*-Value
Age at evaluation, mean (S.D.)	15.42 (1.871)	15.85 (1.897)	0.321
BMI at evaluation, mean (S.D.)	20.72 (3.307)	16.51 (2.958)	<0.001 **
Type of diagnosis, N (%)	CD 9 (27.3) UC 22 (66.7) IBD-U 2 (6.1)	Typical AN 34 (62.9) OSFED 8 (14.8) ARFID 3 (5.6) Atypical AN 9 (16.7)	

Abbreviations: AN, Anorexia Nervosa; ARFID, Avoidant/Restrictive Food Intake Disorder; BMI, Body Mass Index; CD, Crohn’s Disease; UC, Ulcerative Colitis; IBDs, Inflammatory Bowel Disease; IBD-U, Unclassified Inflammatory Bowel Disease; N, number; S.D., standard deviation; OSFED, Other Specified Feeding or Eating Disorder; REDs, Restrictive Eating Disorders. ** ***p*** ≤ 0.001.

**Table 2:** Clinical disease characteristics of patients with IBD (N = 33).

Clinical disease activity at evaluation, N (%)	
remission mild moderate-severe	4 (12.1) 16 (48.5) 13 (39.4)
Endoscopic disease activity at evaluation, N (%)	
remission mild moderate severe	1 (3.0) 7 (21.2) 19 (57.6) 6 (18.2)
Disease duration (months),	
mean (S.D.)	131 (48.67)
Pharmacological treatment, N (%)	
biologics multiple-drug therapy	17 (51.5) 16 (48.5)
Number of steroid cycles,	
mean (S.D.)	1.15 (1.37)
Number of steroid cycles in the previous 6 months,	
mean (S.D.)	0.15 (0.36)
Number of relapses,	
mean (S.D.)	1.18 (1.64)
Number of relapses in the previous 6 months,	
mean (S.D.)	0.12 (0.33)
Number of hospitalizations,	
mean (S.D.)	1.03 (1.10)

Abbreviations: N, number; S.D., standard deviation.

**Table 3:** Comparison of age and BMI at the time of evaluation across the three clinical cohorts (IBD-EDRC>70, IBD-EDRC<70, and REDs).

	COHORT 1 IBD-EDRC>70 (N = 9)	COHORT 2 IBD-EDRC<70 (N = 24)	COHORT 3 REDs (N = 54)	Test 1–2 *p*-Value	Test 2–3 *p*-Value	Test 1–3 *p*-Value
Age at evaluation, mean (S.D.)	16.56 (1.24)	15.00 (1.91)	15.85 (1.90)	0.043 *	0.078	0.332
BMI at evaluation, mean (S.D.)	22.81 (3.36)	19.89 (2.97)	16.51 (2.96)	0.154	<0.001 **	<0.001 **

Abbreviations: BMI, Body Mass Index; EDRC, Eating Disorder Risk Composite; IBD, Inflammatory Bowel Disease; N, number; S.D., standard deviation; REDs, Restrictive Eating Disorders. * *p* ≤ 0.05, ** *p* ≤ 0.001.

**Table 4:** Comparison of EDI-3 composite scale scores among the three clinical cohorts (IBD-EDRC>70 *versus* IBD-EDRC<70 *versus* REDs).

	COHORT 1 IBD-EDRC>70		COHORT 2 IBD-EDRC<70		COHORT 3 REDs		Test 1–2	Test 2–3	Test 1–3
	Mean	S.D.	Mean	S.D.	Mean	S.D.	*p*-Value	*p*-Value	*p*-Value
EDI-IC	77.56	13.73	35.80	23.72	71.31	28.32	0.003 *	<0.001 **	0.961
EDI-IPC	67.89	22.13	46.00	28.99	66.13	27.62	0.111	0.006 *	0.891
EDI-APC	81.78	16.55	59.00	25.90	70.78	24.96	0.008 *	0.009 *	0.271
EDI-OC	77.00	13.04	43.30	27.06	66.87	24.46	0.001 *	<0.001 **	0.303
EDI-GPMC	80.67	11.64	70.19	24.17	74.92	23.41	0.002 *	<0.001 **	0.674

Abbreviations: S.D., standard deviation; APC, Affective Problems Composite; EDRC, Eating Disorder Risk Composite; GPMC, Global Psychological Maladjustment Composite; IBD, Inflammatory Bowel Disease; IC, Ineffectiveness Composite; IPC, Interpersonal Problems; OC, Overcontrol Composite; REDs, Restrictive Eating Disorders. * *p* < 0.05, ** *p* ≤ 0.001.

**Table 5:** Comparison of MAIA-2 results among the three clinical cohorts (IBD-EDRC>70 *versus* IBD-EDRC<70 *versus* REDs).

	COHORT 1 IBD-EDRC>70		COHORT 2 IBD-EDRC<70		COHORT 3 REDs		Test 1–2	Test 2–3	Test 1–3
	Mean	S.D.	Mean	S.D.	Mean	S.D.	*p*-Value	*p*-Value	*p*-Value
MAIA-2 Noticing	3.11	1.06	2.40	1.27	2.58	1.13	0.253	0.832	0.273
MAIA-2 Not-Distracting	2.33	1.04	2.45	0.92	2.644	1.07	0.518	0.776	0.371
MAIA-2 Not-Worrying	2.06	1.20	2.99	1.15	2.096	1.07	0.050 *	0.002 *	0.991
MAIA-2 Attention Regulation	2.31	0.91	3.00	0.91	2.111	1.06	0.126	<0.001 **	0.509
MAIA-2 Emotional Awareness	3.00	1.35	3.17	1.07	2.537	1.39	0.965	0.092	0.261
MAIA-2 Self-Regulation	1.86	1.24	2.56	1.08	1.276	1.05	0.190	<0.001 **	0.132
MAIA-2 Body Listening	1.92	1.16	2.69	1.08	1.313	1.17	0.142	<0.001 **	0.154
MAIA-2 Trusting	1.92	0.97	3.69	1.02	1.739	1.38	0.004 *	<0.001 **	0.641

Abbreviations: S.D., standard deviation; IBD, Inflammatory bowel disease; EDRC, Eating Disorder Risk Composite; REDs, Restrictive Eating Disorders. * *p* < 0.05, ** *p* ≤ 0.001.

**Table 6:** Spearman’s correlation between disease features and MAIA-2 subscales in IBDs at risk for eating disorders (IBD-EDRC>70).

Disease Features	Noticing		Not Distracting		Not Worrying		Attention Regulation		Emotional Awareness		Self- Regulation		Body Listening		Trusting	
	ρ	*p*	ρ	*p*	ρ	*p*	ρ	*p*	ρ	*p*	ρ	*p*	ρ	*p*	ρ	*p*
Age at diagnosis	−0.148	0.704	−0.261	0.498	−0.205	0.596	−0.204	0.598	0.035	0.928	−0.013	0.973	−0.210	0.588	−0.180	0.643
BMI at questionnaire	−0.661	0.053	0.418	0.262	0.504	0.166	−0.536	0.137	−0.593	0.092	−0.075	0.847	−0.286	0.456	0.118	0.762
Number of relapses	−0.774	0.014 *	−0.014	0.972	0.125	0.749	−0.525	0.147	−0.308	0.420	−0.138	0.723	−0.458	0.215	−0.209	0.589
Relapses (last 6 months)	−0.138	0.724	−0.413	0.270	−0.138	0.723	0.000	1.000	0.418	0.263	0.344	0.365	0.069	0.860	0.208	0.591
Steroid cycles	−0.353	0.352	0.247	0.522	−0.292	0.445	−0.565	0.113	0.152	0.696	0.022	0.955	−0.013	0.973	0.307	0.422
Steroid cycles (last 6 months)	0.104	0.790	−0.208	0.591	0.052	0.894	0.208	0.591	0.421	0.259	0.676	0.046 *	0.365	0.334	0.524	0.147
Hospitalizations	−0.230	0.552	−0.330	0.386	−0.352	0.354	−0.240	0.534	0.091	0.816	−0.125	0.749	−0.347	0.361	−0.277	0.470
Disease duration	−0.416	0.265	0.580	0.102	0.030	0.940	−0.697	0.037 *	−0.443	0.233	−0.756	0.018 *	−0.443	0.232	0.110	0.778

Abbreviations: ρ, Spearman’s correlation coefficient; *p*, *p*-value. * *p* < 0.05.

## Data Availability

The datasets used and analysed in the current study are available from the corresponding author on reasonable request due to privacy.
